# Small cell carcinoma of uterine cervix: A case report

**DOI:** 10.22088/cjim.15.3.546

**Published:** 2024-08-01

**Authors:** Danial Fazilat-Panah, Mohammad Hassan Emranpour, Babak PeyroShabany, Sara Rasta, Maedeh Alsadat Fatemi, Zeinab Nazari, Yavar Rajabzadeh

**Affiliations:** 1 Cancer Research Center, Babol University of Medical Sciences, Babol, Iran; 2 Radiation Oncology Clinic, Babol, Iran; 3 Department of Internal Medicine, Sabzevar University of Medical Sciences, Sabzevar, Iran; 4 Non-communicable Diseases Research Center, Alborz University of Medical Sciences, Karaj, Iran; 5 Mazandaran University of Medical Sciences, Sari, Iran

**Keywords:** Small cell carcinoma, Uterine cervix, Radiation therapy, Chemotherapy

## Abstract

**Background:**

Small cell carcinoma of cervix (SCCC) is a rare disease. SCCC is highly invasive and prone to distant metastatic spread and lymph node involvement. Here we aim to present a patient and her treatment.

**Case Presentation:**

We report 47-year-old patient with history of breast cancer manifesting with abnormal vaginal bleeding diagnosed with SCCC. Patient underwent radical hysterectomy and bilateral salpingo-oophorectomy. Then, she received adjuvant chemoradiation postoperatively.

**Conclusion:**

Small cell carcinoma of cervix is an aggressive form of cervical cancer with poor prognosis. Optimal treatment remains unsettled.

An estimated 13960 new cervical cancers and 4,310 cervical cancer deaths will occur in the United States in 2023 according to American cancer society (1). Cervical cancer in still one of the most common cancers among females, taking the fourth place after breast, colorectal, and lung cancer (2) The majority of new cases and deaths (approximately 85% and 90%, respectively) presents itself in low-income regions or among people from socioeconomically weaker sections of society (3). 

About 90% of cervical cancer patients are squamous cell carcinoma. Small cell carcinoma of the cervix (SCCC) is a uncommon entity comprising approximately 2–5% of uterine cervix malignancies (4-6). SCCC have histological features that resemble small cell neuroendocrine neoplasms of the lung cancer (7,8). Small-cell lung cancer represents about 15% of all lung cancers and is identified by an abnormally high proliferative index, strong propensity for early metastasis and poor prognosis. SCCC is highly invasive and inclined to distant metastatic spread and lymph node involvement causing poorer prognosis than other types of cervical cancer (9,10). Its clinical manifestations and presentations are similar to those of other cervical cancers (11).

## Case Presentation

Our patient is a 47 year-old lady presented with a 2 month history of abnormal vaginal bleeding and postcoital bleeding. Patient also complained of mild abdominal discomfort but no other symptoms including gastrointestinal and urinary symptoms were reported. Unfortunately, the patient had not done regular Pap smear test over the years but did not have prior abnormal Pap smear. On past medical history, she had breast cancer, invasive ductal carcinoma stage T2N3, about 6 years ago. She then underwent lumpectomy and axillary lymph node dissection.

Subsequently she received chemotherapy followed by radiotherapy and later due to premenopausal status tamoxifen and GnRh agonist was prescribed. On physical examination for these recent symptoms the patient had a 3cm mass in cervix.

**Figure 1:**
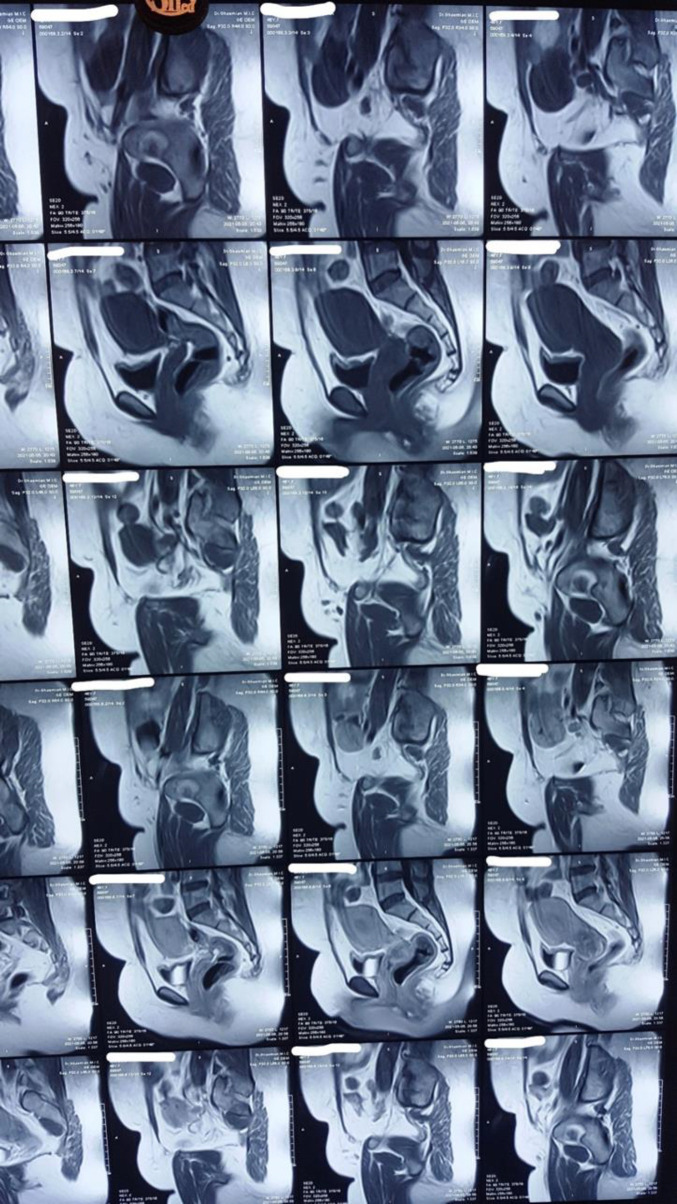
Images show dilatation of cervical canal with mucosal irregularity and thickening

**Figure 2:**
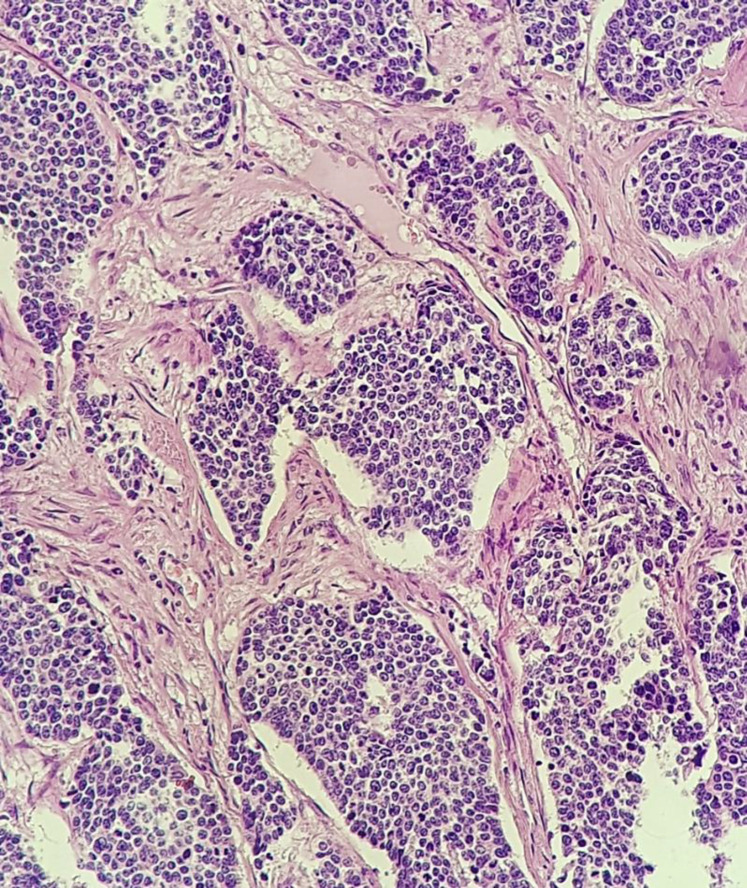
Sections from cervical uterine mass reveal malignant epithelial neoplasm composed of solid sheets and nests of atypical cells having small vesicular to hyperchromatic nuclei and frequent mitoses

**Figure 3:**
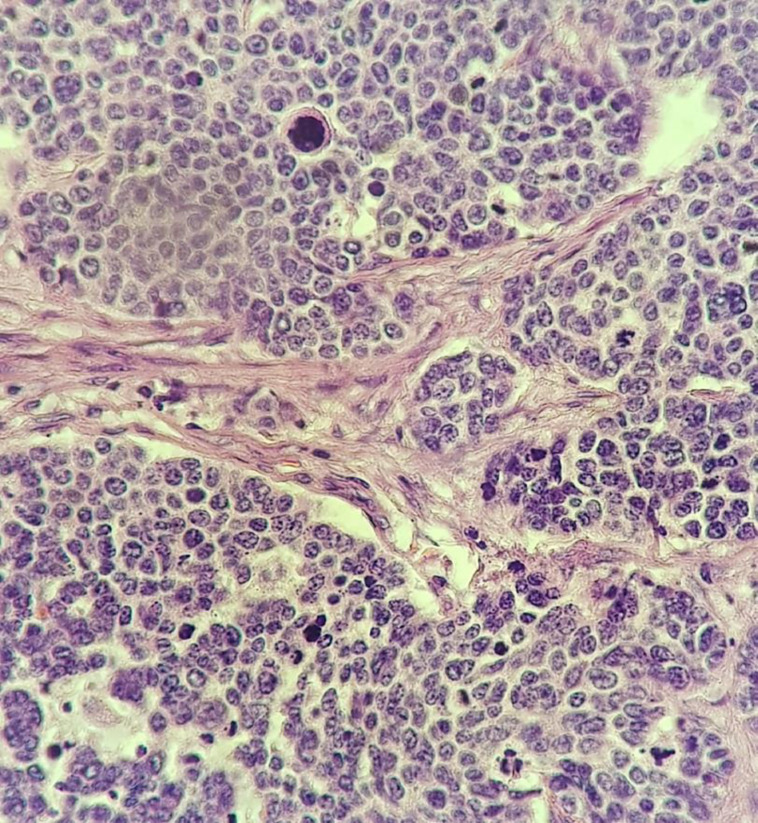
Sections from cervical uterine mass reveal malignant epithelial neoplasm composed of solid sheets and nests of atypical cells having small vesicular to hyperchromatic nuclei and frequent mitoses

An abdominal sonography revealed a 7*7 mm hypoechoic lesion in uterine cervix. MRI was also done which reported cervical canal dilatation with mucosal irregularity and thickening (illustrated in figure 1).Two lymph nodes with short axis diameter (SAD) of 11 & 13 mm in right side of pelvis and mild fat stranding in right side of paracervical region. Core needle biopsy was done which reported to be small cell carcinoma of cervix. Radical hysterectomy and bilateral salpingo-oophorectomy was done for the patient along with pelvic lymphadenectomy Pathology is as follows (microscopic view is demonstrated in figures 2 and 3). Histology is identified as poorly differentiated small cell non-keratinized carcinoma with horizontal extent about 2 cm, depth of stromal invasion 7 mm, detected perineural and lymphovascular invasion and vaginal wall, uterine corpus, both parametria and adnexae free from tumor. In addition, 18 out of 19 dissected lymph nodes were involved and surgical margins were free.

**Figure 4:**
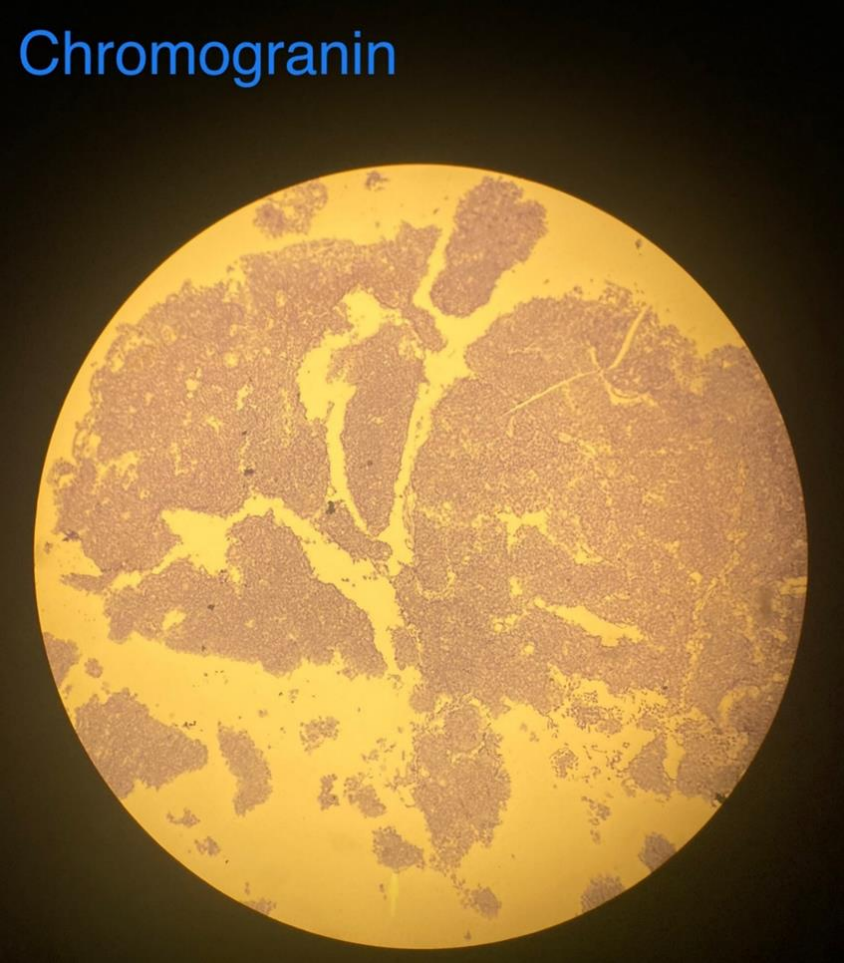
This figure shows positive results for chromogranin

**Figure 5:**
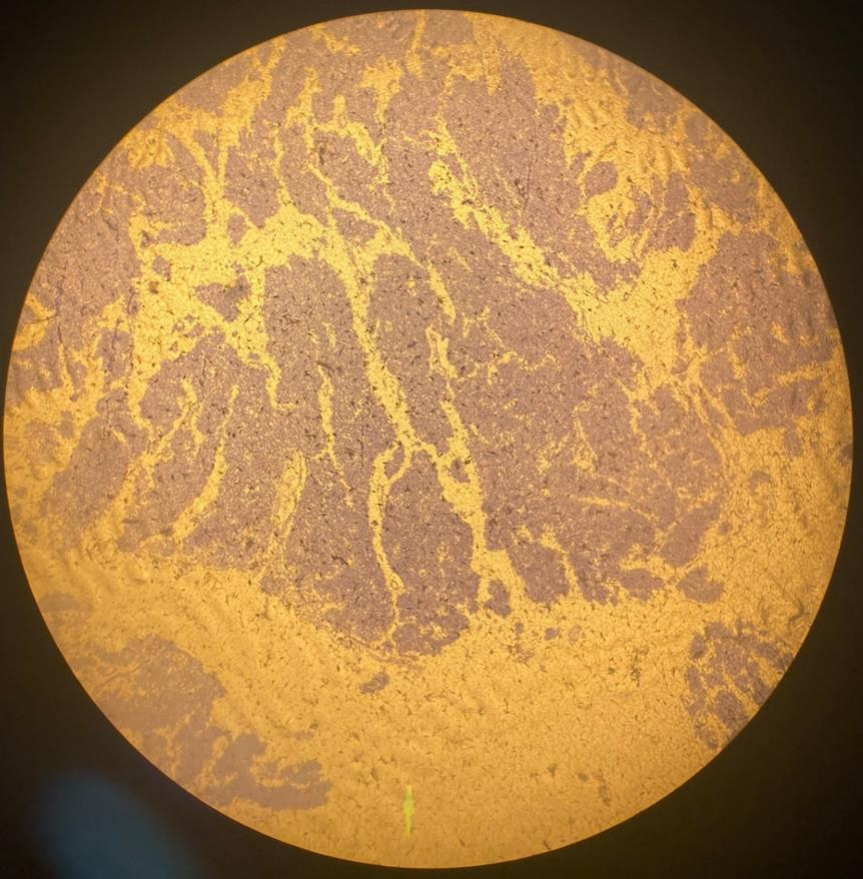
This photo shows positive results for synaptophysin stain

**Figure 6:**
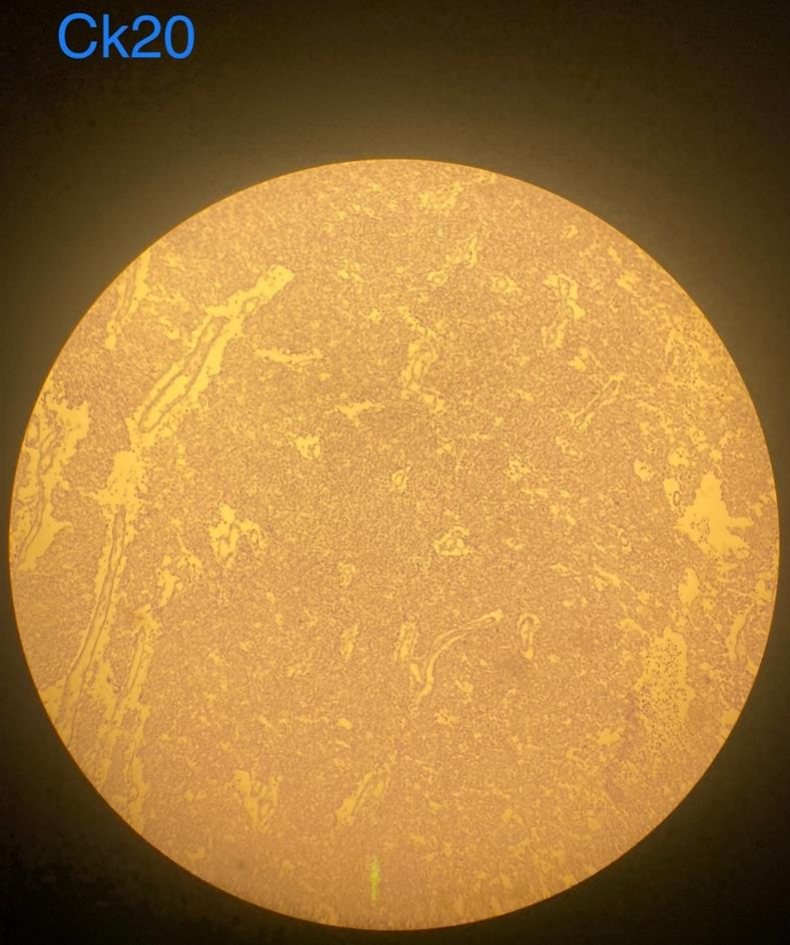
This figure shows negative results for cytokeratin 20 stain

Immunohistochemistry (IHC) for synaptophysin, chromogranin and cytokeratin 20 was used to establish the diagnosis of small cell carcinoma (figures 4-6). Patient was referred to our hospital oncology ward after surgery. After surgical wound healing adjuvant treatment with 6 cycles of cisplatin and etoposide and concurrent radiation therapy (starting with cycle 2 prescribed at 4600 cGy/23 fractions) commenced according to protocols for small cell lung cancer regimen. Intravaginal brachytherapy was also instrumented after completion of external beam radiotherapy. At the time of writing this paper, patient is in follow-up and free of tumor recurrence and metastasis. Diagnosis of cervix carcinoma highlights the value of continued follow-up of breast cancer patients.

## Discussion

SCCC is a relatively rare subtype of cervical malignancy. It can be categorized as small cell carcinoma of extrapulmonary origin but nowadays is identified as a clinicopathological disease with biological behavior and prognosis dissimilar to small-cell lung carcinoma (SCLC) (12). Small-cell carcinomas of extrapulmonary origin parallel small-cell carcinomas of the lung and are comprised of small tumoral cells that have scant cytoplasm, small round to oval nuclei, and high mitotic index; they frequently display neuroendocrine features (5). Almost all SCCC are immunoreactive for keratin and epithelial membrane antigen and at least one marker of neuroendocrine differentiation is expressed in 88 to 100 percent of cases (including neuron-specific enolase, synaptophysin, CGA and CD 56) (13). 

Overall, these tumors are usually aggressive, with early spreading and frequent recurrences. Despite the fact that chemotherapy seems to be an effective therapeutic method like the case in SCLC, surgery and radiotherapy may also have an important role depending on the stage or primary site (14).

A large case series of extrapulmonary small cell carcinomas from England identified 76 cervix cases out of 1618 (4.69 %) (15). Other small series have reported different numbers. For example, a case series from South Korea published in 2004 reported a 29% rate for a cervical site (16). As previously stated, presentation and clinical manifestations of SCCC is the same as other cervical cancers.

Multiple parameters have been noted as prognostic. In a review of 188 patients, writers concluded that use of adjuvant chemotherapy or chemoradiation linked with higher survival in small cell cervical cancer patients (17). In another review of 290 patients from the surveillance, epidemiology, and end result database on multivariate analysis, age, stage, and race were prognostic for survival in women with small cell carcinoma (18). Other series have listed advanced disease (19, 20), smoking (20), lymph node metastasis (21) and hematogenous metastasis (22) as prognostic factors.

Regarding its etiologic factors, a recent meta-analysis of 143 studies has revealed HPV-16 and HPV-18 to be the cause of most small cell carcinomas of cervix (23). Different modalities are implemented for treatment by oncologists. Combination therapy by surgical resection and postoperative chemotherapy or chemoradiation for early stage resectable disease, definitive chemoradiation for locoregionally advanced disease and palliative chemotherapy for patients with metastatic disease are among the options. Chemotherapy is usually based on small cell lung cancer regimens (24).

 In a recent systematic review of literature, Tempfer et al. have pointed to similar trends in practice and concluded that cisplatin/carboplatin with etoposide alone or in combination with other agents is the most common regimen (25). Since SCCC is a rare disease, most series have small numbers and no prospective trial has been done to this date, data are limited to guide decision-making and there is no consensus as to optimal management (13). Treatment generally considers the treatment options for cervical cancer, particularly chemotherapy, which have been largely extrapolated from the experience with small cell lung cancer (9).

In a review of 100 extrapulmonary, small cell carcinoma authors concluded that definitive chemoradiation was associated with improved outcomes. Additionally, prophylactic cranial irradiation improved overall survival but the benefit was less than SCLC (26). In conclusion, small cell carcinoma of cervix is an aggressive form of cervical cancer with poor prognosis. Prognosis is poor and optimal treatment remains unsettled.
